# Synthesis of Sulfonated Polyphenylene Block Copolymers via In Situ Generation of Ni(0)

**DOI:** 10.3390/polym15061577

**Published:** 2023-03-22

**Authors:** Vikrant Yadav, Farid Wijaya, Hyejin Lee, Byungchan Bae, Dongwon Shin

**Affiliations:** 1 Fuel Cell Laboratory, Korea Institute of Energy Research, Daejeon 34129, Republic of Korea; 2 Hydrogen Energy Engineering, University of Science and Technology, Daejeon 34113, Republic of Korea

**Keywords:** sulfonated polyphenylene, block copolymer, nickel-catalyzed cross-coupling reaction, Ni(0) regeneration, proton exchange membrane

## Abstract

Proton exchange membranes (PEMs) fabricated from sulfonated polyphenylenes (sPP) exhibit superior proton conductivity and electrochemical performance. However, the Ni(0) catalyst required for Colon’s cross-coupling reaction for the synthesis of sPP block copolymers is expensive. Therefore, in this study, we generated Ni(0) in situ from an inexpensive Ni(II) salt in the presence of the reducing metal Zn and NaI. The sPP block copolymers were synthesized from neopentyl-protected 3,5- and 2,5-dichlorobenzenesulfonates and oligo(arylene ether ketone) using the catalyst NiBr_2_(PPh_3_)_2_. The block copolymers synthesized using our strategy and the Ni(0) catalyst exhibited comparable polydispersity index values and high molecular weights. Thin, transparent, and bendable PEMs fabricated using selected high-molecular-weight sPP block copolymers synthesized via our strategy exhibited similar proton conductivities to those of the block copolymers synthesized using the Ni(0) catalyst. We believe that our strategy will promote the synthesis of similar multifunctional block copolymers.

## 1. Introduction

Proton exchange membranes (PEMs) are essential components of energy storage and conversion devices. The performance and cost of these devices are highly dependent on the properties and costs of PEMs. Perfluorosulfonic acid (PFSA) membranes have received considerable attention owing to their excellent electrochemical performance and durability; however, these membranes have several drawbacks such as high cost, poor environmental compatibility, narrow operating temperature range, and high gas permeability [1,2,3]. In order to overcome these limitations, research efforts should be directed toward the development of high-performance and low-cost alternative polymer electrolytes.

Aromatic polymers can overcome the limitations of PFSA ionomers because of their diverse molecular structures, facile synthesis, and low cost. Several sulfonated aromatic polymers have been investigated as membrane materials and outperformed PFSA membranes in terms of electrochemical performance [4,5,6]. However, statistical sulfonated aromatic copolymers exhibit chemical instability, dependence of relative humidity (RH) on the proton conductivity, and excessive swelling behavior [7,8]. Hence, multiblock copolymers have been designed by incorporating rigid hydrophilic blocks such as sulfonated polyphenylenes to overcome the shortcomings of existing aromatic ionomers [2,3,9,10]. Polyphenylenes are the most promising alternative candidates for the development of PEMs. Sulfonated polyphenylene exhibits chemical stability and mechanical toughness in the polymer backbone [1,4,11,12]. The structure of polyphenylene guarantees appreciable chemical stability even at elevated temperatures because of the absence of vulnerable heteroatoms in the backbone and the high aromatic C–C bond dissociation energy. Block copolymers are self-assembled, structurally interesting materials in which the morphology and domain size are harmonized at the nanoscale level. This is because ionic and nonionic blocks facilitate ion transport in a reliable order.

Colon’s Ni-catalyzed cross-coupling reactions are one of the most promising approaches for synthesizing polyphenylenes and can be applied to various polymers using various starting materials [13,14]. The zerovalent Ni complex Ni(COD)_2_ is one of the best catalysts for the synthesis of polyphenylenes, but its high cost could undermine the economic benefits of hydrocarbon-based membranes. In contrast, inexpensive Ni(II) complexes have been proven effective in catalyzing polymerization to obtain high-molecular-weight polymers [15]. In situ generation of Ni(0) from Ni(II) is a key limitation associated with the use of Ni(II) complexes. The use of a strong reducing metal in Colon’s C–C coupling reaction system promotes the reduction in Ni(II) to Ni(0). In addition, the metal can act as an acceptor in the presence of halides. Therefore, using the right reducing metal is important. Furthermore, an alkali metal salt such as NaI would act as a chaotropic agent and an additive weak ligand that neutralizes the acidity generated in Colon’s C–C coupling reaction and would supply iodide ions for a halide exchange [16,17].

Therefore, we synthesized sulfonated polyphenylene-based multiblock copolymers using a Ni(II) salt in the presence of the strong reducing metal Zn and the alkali metal salt NaI. Furthermore, we investigated the effect of the Ni(II) equivalents in relation to those of total chlorine and optimized the polymerization conditions. We also observed the effect of the sulfonated monomer ratio under the optimized polymerization conditions on the resultant polymer molecular weight. Moreover, the resultant polymers and membranes were compared with those produced by Ni(0)-complex-catalyzed polymerization.

## 2. Materials and Methods

### 2.1. Materials

Bis(triphenylphosphine)nickel(II) dibromide (NiBr_2_(PPh_3_)_2_, 99%), potassium carbonate (K_2_CO_3_, ≥99%), calcium carbonate (CaCO_3_, ≥99%), bis(1,5-cyclooctadiene)nickel(0) (Ni(COD)_2_), sodium iodide (NaI, ≥99%), lithium bromide (≥99%), sodium bicarbonate (NaHCO_3_, ≥99.7%), pyridine (≥99%), anhydrous *N,N*-dimethyl acetamide (DMAc, 99.8%), anhydrous 1-methyl-2-pyrrolidone (NMP, 99.5%), anhydrous dichloromethane (DCM, ≥99.8%), anhydrous diethyl ether (≥99.7%), deuterochloroform (CDCl_3_, 99.8 atom% D containing 0.03% *v*/*v* TMS), hexadeuterodimethyl sulfoxide (*d*_6_-DMSO, 99.8 atom% D containing 0.03% *v*/*v* TMS) were purchased from Sigma-Aldrich (St. Louis, MO, USA). 4,4-Dichlorobenzophenone (DCBP, >99%), 2,2-bis(4-hydroxyphenyl)hexafluoropropane (6F-BP, >98%), 2,5-dichlorobenzene sulfonyl chloride (*p*DCBS, >98%), 3,5-dichlorobenzene sulfonyl chloride (*m*DCBS, >98%), 2,2-dimethyl propanol (>98%), and 2,2-bipyridine (BPy, >99%) were purchased from Tokyo Chemical Industry (Chuo-ku, Japan). Methyl alcohol and ethyl alcohol were purchased from Samchun Pure Chemicals (Seoul, Republic of Korea). HPLC-grade dimethylacetamide (DMAc), extra pure-grade HCl, NMP, DMAc, *n*-hexane, DCM, and NaHCO_3_ were purchased from Duksan Chemicals (Ansan, Republic of Korea). The obtained materials were used as received unless otherwise stated.

Potassium carbonate and calcium carbonate were vacuum-dried at 150 °C for 12 h before use. Triphenylphosphine (PPh_3_, Sigma-Aldrich, 99%) was recrystallized from *n*-hexane and dried under reduced pressure at 50 °C for 24 h. Zinc powder (<150 μm, 99.995%, Sigma-Aldrich) was stirred in HCl (1 mol/L in ether; TCI) for 10 min, filtered, washed with anhydrous diethyl ether (five-fold), and dried in Abderhalden’s drying pistol at 150 °C.

### 2.2. Synthesis of Neopentyl 2,5-Dichlorobenzenesulfonate and Neopentyl 3,5-Dichlorobenzenesulfonate

Neopentyl 2,5-dichlorobenzene sulfonate (*p*NDCBS) and neopentyl 3,5-dichlorobenzene sulfonate (*m*NDCBS) were synthesized according to a previously reported method [3]. The ^1^H NMR spectra of *p*NDCBS and *m*NDCBS, along with those of the starting materials, are presented in Figure S1. The chemical shift values were in good agreement with the predicted values for the proposed structure.

^1^H NMR of *m*DCBS (in CDCl_3_ at 600 mHz): *δ* = 7.72 (t, 1H) and 7.92 (d, 2H); *m*NDCBS (in CDCl_3_ at 600 mHz): *δ* = 0.94 (s, 9H), 3.75 (s, 2H), 7.63 (t, 1H), and 7.78 (d, 2H). The yield of *m*NDCBS was 89%.

^1^H NMR of *p*DCBS (in CDCl_3_ at 600 mHz): *δ* = 7.58 (dd, 1H), 7.63 (dd, 1H), and 8.14 (d, 1H); *p*NDCBS (in CDCl_3_ at 600 mHz): *δ* = 0.96 (s, 9H), 3.78 (s, 2H), 7.51 (dd, 1H), 7.55 (dd, 1H), 8.07 (d, 1H). The yield of *p*NDCBS was 92%.

### 2.3. Synthesis of Hydrophobic Cl-End-Capped OAEK Oligomer

The hydrophobic block of oligo(arylene ether ketone) (OAEK) with a repeating unit length of five (X5) units was prepared using 4,4-dichlorobenzophenone and 2,2-bis(4-hydroxyphenyl)hexafluoropropane monomers. Briefly, a 100-mL four-neck double-jacketed flask (Radleys, Essex, UK) equipped with a reflux condenser, overhead stirrer, and argon inlet/outlet was charged with 6F-BP (5.0 g, 14.87 mmol) and DCBP (4.84 g, 17.85 mmol) at 120 °C. The required DMAc was added to the mixture to obtain a 20 wt% transparent homogeneous solution. CaCO_3_ (8.93 g, 89.23 mmol) was added to this solution and the temperature was increased to 150 °C, following which K_2_CO_3_ (3.08 g, 22.31 mmol) was added to initiate the reaction. The reaction progress was examined by periodic gel permeation chromatography (GPC). Next, the Cl-end-capping agent DCBP (0.45 g, 1.78 mmol) was added to the reaction mixture, and the reaction was allowed to proceed for 6 h to ensure full end-capping. The mixture was filtered through a 0.45-µm membrane (Merck Millipore, Burlington, MA, USA) under reduced pressure, and the filtrate was precipitated in methyl alcohol. The white precipitate was washed several times with ethyl alcohol and vacuum-dried for 12 h at 80 °C.

### 2.4. Synthesis of sPP Block Copolymers via Ni(II)-Catalyzed Colon’s Cross-Coupling Reaction

**Scheme 1:**
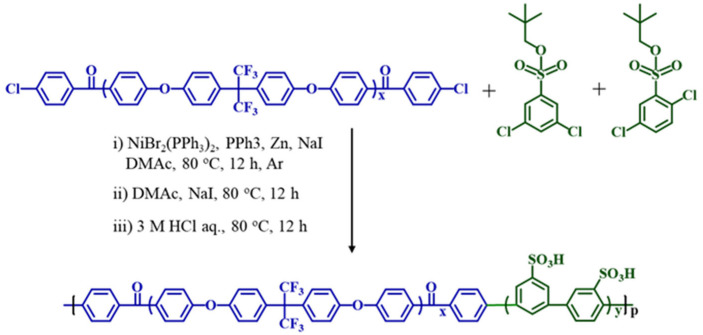
Schematic of the synthesis of an sPP block copolymer using in-situ-generated Ni(0).

The block copolymerization reaction of neopentyl-protected monomers (2,5/3,5-dichlorobenzene sulfonate) and hydrophobic OAEK oligomer (X5) (Scheme 1) in the presence of the NiBr_2_(PPh_3_)_2_ catalyst was investigated under different conditions. The length of the hydrophilic block (Y) was determined by adjusting the molar equivalents of neopentyl-protected dichlorobenzene sulfonate monomers to afford a 2.50 meq/g IEC of the final block copolymer. The equivalents of NiBr_2_(PPh_3_)_2_ were varied in relation to the total chlorine equivalents. A 1:1 ratio of *m*NDCBS and *p*NDCBS, along with 0.030, 0.050, 0.065, and 0.075 equivalents of NiBr_2_(PPh_3_)_2_, were used to afford sPP30@1:1, sPP50@1:1, sPP65@1:1, and sPP75@1:1, respectively. Similarly, a 1:4 ratio of *p*NDCBS to *m*NDCBS and 0.065 equivalents of NiBr_2_(PPh_3_)_2_ afforded sPP65@1:4. In all the experiments, the molar ratios of catalyst to ligand (1:10), catalyst to Zn (1:30), and catalyst to NaI (1:1) were kept constant, and the solution concentration was adjusted to 10 wt% considering the theoretical yield of the polymer. sPP30@1:1 refers to sulfonated polyphenylene, which was prepared in the presence of 0.030 equivalents of NiBr_2_(PPh_3_)_2_ to the total terminal chlorine and a hydrophilic phenylene ratio (*p*NDCBS: *m*NDCBS) of 1:1. In the case of sPP(COD)@1:1, 1.25 equivalents of Ni(COD)_2_ to the total terminal chlorine were used. Considering the polymerization of sPP65@1:1 as an example, the typical polymerization process is as follows: To a previously dried 100-mL four-neck double-jacketed flask fitted with an oil circulator and overhead mechanical stirrer, a mixture of NiBr_2_(PPh_3_)_2_ (0.22 g, 0.30 mmol), PPh_3_ (0.79 g, 3.00 mmol), Zn (0.59 g, 9.00 mmol), and NaI (0.05 g, 0.30 mmol) was added at 80 °C under continuous Ar flow (40 cc/min). DMAc (6.58 g) was added to this mixture. Next, a solution of *p*NDCBS (0.63 g, 2.12 mmol), *m*NDCBS (0.63 g, 2.12 mmol), and the OAEK oligomer (1.23 g, 0.38 mmol) in DMAc (11.39 g) was injected into the flask using a syringe. The reaction mixture was allowed to react at the same temperature for 12 h. Then, the reaction mixture was diluted and precipitated into a large excess of 6 M HCl/MeOH. The obtained white fibrous precipitate was washed with methanol, 1 M NaHCO_3_, DI water, and methanol and vacuum-dried at 80 °C.

### 2.5. Synthesis of sPP Block Copolymers via Ni(0)-Catalyzed Colon’s Cross-Coupling Reaction

For a comparative study, the two polymers sPP(COD)@1:1 and sPP(COD)@1:4 were synthesized with a target IEC of 2.50 meq/g using the Ni(COD)_2_ catalyst. The molar equivalents of *p*NDCBS and *m*NDCBS used for sPP(COD)@1:1 was 1:1, whereas that for sPP(COD)@1:4 was 1:4. Other reaction conditions were similar in both cases: The catalyst equivalents were 1.25 to those of total chlorine, the catalyst to ligand ratio was 1:2, and the solution concentration was adjusted to 20 wt% using the theoretical yield of the polymer. A typical polymerization procedure for sPP(COD)@1:1 is as follows: To a previously dried four-neck double-jacketed flask connected to an oil circulator and overhead mechanical stirrer, OAEK oligomer (1.43 g, 0.47 mmol), *p*NDCBS (0.794 g, 2.67 mmol), and *m*NDCBS (0.79 g, 2.67 mmol) were added at 80 °C under Ar flow (40 cc/min), following which NMP (7.28 g) was added. To this mixture, BPy (2.55 g, 14.54 mmol), 2.76 g of NMP, and Ni(COD)_2_ (2.00 g, 7.27 mmol) were successively added, following which the reaction was allowed to proceed at the same temperature for 4 h. The viscous solution was diluted by adding NMP (4 g). After the mixture was cooled, it was poured into a 6 M HCl/MeOH solution, filtered, and washed with methanol. The obtained brown reddish precipitate was washed with 1 M NaHCO_3_, water, and methanol and vacuum-dried at 80 °C.

### 2.6. Deprotection and Protonation of sPP Block Copolymers

sPP block copolymers with reasonable molecular weights were considered for deprotection and protonation reactions. Briefly, a solution of sPP block copolymer (2.00 g, 0.38 mmol) in DMAc (18.00 g) was added to a 50-mL round-bottom flask fitted with a reflux condenser, magnetic stirrer, and Ar inlet/outlet at 80 °C. NaI (4.07 g, 27.13 mmol) was added to this solution, and the reaction mixture was allowed to react at the same temperature for 12 h under Ar flow. Next, the reaction mixture was poured into excess ethanol, filtered, and washed with ethanol. The filtrate was then washed with chloroform to remove any unprotected sPP block copolymer. The samples were washed with ethanol and vacuum-dried at 80 °C.

The dried precipitate was further treated with 3 M HCl/H_2_O in a closed vessel equipped with a magnetic stirrer and thermometer at 80 °C for 12 h to ensure the complete protonation of the deprotected polymer. Subsequently, the polymer was washed with DI water until a neutral pH was reached and then vacuum-dried at 80 °C.

### 2.7. Preparation of sPP Block Copolymer Membranes

The sPP block copolymer solution was prepared using DMAc, and the resultant homogeneous transparent solution was filtered through a 0.45-µm membrane and degassed. The degassed solution was cast onto a flat glass plate, dried at 70 °C for 24 h, and then further vacuum-dried at 100 °C for 12 h to ensure the complete evaporation of DMAc. The membranes were treated with 3 M HCl/H_2_O for 12 h at 80 °C in a closed vessel, washed with DI water until a neutral pH was achieved, and then dried at 30 °C for 24 h. The thicknesses of the membranes were adjusted to 35 µm.

### 2.8. Molecular Weight Measurements

The molecular weights of the oligomer and the prepared block copolymers were analyzed using a gel permeation chromatography (GPC) instrument equipped with a Younglin YL 9112 isocratic pump and YL 9120 UV–visible detector (Younglin Instruments, Anyang, Republic of Korea). Shodex SB-803HQ and Shodex KF-805L columns were used for polymer and oligomer analyses, respectively. DMAc containing 0.01 M LiBr was used as the eluent. Molecular weights were confirmed using standard polystyrene samples.

### 2.9. Structural Analysis

^1^H NMR spectra were recorded on an AVANCE III (600 MHz) spectrometer (Bruker BioSpin, Billerica, MA, USA) in CDCl_3_ or *d*_6_-DMSO using tetramethylsilane (TMS) as the internal reference standard.

### 2.10. Water Uptake, Swelling Ratio, and Conductivity Measurements for Membranes

For the measurements, the membrane samples were dried at 120 °C for 24 h. Then, the dried membrane samples were immersed in DI water for 24 h at 25 °C and 3 h at 80 °C. The water uptake, as well as the in-plane and through-plane swelling behavior of the membranes in protonated form, were analyzed in DI water using the following equations:

(1) $$\mathrm{Water}\;\mathrm{uptake}\;(\%)=\frac{W_{\mathrm{wet}}-W_{\mathrm{dry}}}{W_{\mathrm{dry}}}\times 100$$

(2) $$\mathrm{In-plane}\;\mathrm{swelling}\;(\%)=\frac{A_{\mathrm{wet}}-{\;A}_{\mathrm{dry}}}{A_{\mathrm{dry}}}\times 100$$

(3) $$\mathrm{Through-plane}\;\mathrm{swelling}\;(\%)=\frac{T_{\mathrm{wet}}-{\;T}_{\mathrm{dry}}}{T_{\mathrm{dry}}}\times 100$$

where W_wet_ and W_dry_ are the weights of the membrane samples in wet and dry states, respectively; A_wet_ and A_dry_ are the areas of the membrane under wet and dry conditions, respectively; and T_wet_ and T_dry_ are the wet and dry membrane thicknesses, respectively. Water vapor uptake was recorded on a TGA Q5000 instrument at 30, 50, and 70% RH conditions at 80 °C. 

In-plane proton conductivity was measured in four-probe conductivity cells (WonATech, Seoul, Republic of Korea) using an electrochemical impedance analyzer. The analysis was conducted in the frequency range of 10^−1^–10^5^ Hz at an amplitude of 100 mV using a Solartron 1287 electrochemical interface and Solartron 1260 impedance/gain-phase analyzer. Proton conductivity was calculated using the following equation:

(4) $$\sigma \;(S/\mathrm{cm})=\frac{D}{L\;\times \;T\;\times \;R}\times 100$$

where D, L, and T are the distance between the electrodes, width of the sample, and thickness of the sample, respectively, and R is the resistance obtained by extrapolating the Nyquist plot to the x-intercept in the frequency range of 10^−1^–10^5^ Hz.

## 3. Results and Discussion

### 3.1. Synthesis of Hydrophobic Cl-End-Capped OAEK Oligomer

**Figure 1:**
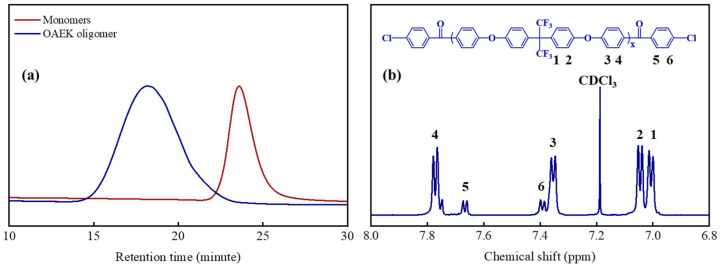
(**a**) Gel permeation chromatography (GPC) profile and (**b**) ^1^H NMR spectra of the Cl-end-capped OAEK oligomer recorded in CDCl_3_.

A Cl-end-capped OAEK was synthesized by a polycondensation reaction at elevated temperatures. The end-group functionality and the number of repeating units were controlled by adjusting the feed monomer ratio. However, the ether–ether exchange reaction during the polycondensation at elevated temperatures is a serious limitation [18]. During the nucleophilic step of oligomerization at high reaction temperatures, randomization of repeating unit sequences occurs via ether–ether exchange reactions. The GPC profile of the OAEK oligomer with a length of X5 and the corresponding ^1^H NMR spectra are presented in Figure 1. The chemical shift values were in good agreement with the predicted values for the proposed structure, suggesting the absence of side reactions such as ether–ether exchange reactions. Here, the use of CaCO_3_ functioned well in inhibiting the ether–ether exchange reaction [19]. In addition, the number of repeating units calculated from the integral ratio of protons of the repeating units and protons of the end group was nearly five. 

^1^H NMR (in CDCl_3_ at 600 mHz): *δ* = 7.08 (20H, d), 7.12 (20H, d), 7.43 (20H, d), 7.46 (4H, d), 7.74 (4H, d), and 7.85 (20H, d). The yield of the hydrophobic OAEK oligomer was 84%.

### 3.2. Block Copolymer Synthesis Using NiBr_2_(PPh_3_)_2_ with the Reducing Metal Zn

Among the various parameters affecting the polycondensation, the following factors are crucial: (i) anhydrous conditions must be maintained in the presence of Ni(0) as water reduces the aryl halide to arene, thereby inhibiting polymerization and deactivating the catalyst; (ii) oxygen in the presence of triphenylphosphine deactivates the catalyst. Nevertheless, the reaction system is highly reducing in nature, but nickel oxide is highly stable and cannot be reduced under these conditions; and (iii) the quality and surface area of Zn.

The choice of the catalyst is important for a high polymeric yield and high molecular weight. Here, the Ni(II) catalyst acts as a color indicator. The catalytic mixture turns red from green upon Ni(II) reduction. The reaction mixture turns greenish yellow upon the addition of polycondensation monomers, which then turn red in color. This phenomenon confirms the progress of the reaction, whereas a persistent greenish color indicates that the Ni complex is not reduced to Ni(0), and a greyish color indicates total catalyst deactivation. Moreover, the ligand system is critical. Aryl transfer from triphenylphosphine (PPh_3_) to Ni is a serious limitation during polymerization [17,20]. The reaction temperature and ligand concentration also have profound effects on this phenomenon [21]. Tonozuka et al. used bipyridyl as a co-ligand with PPh_3_ to suppress such reactions and reported a high polymerization yield and high-molecular-weight polymer by finely tuning the ratio of the ligand and co-ligand with respect to the catalyst [12]. In this study, the NiBr_2_(PPh_3_)_2_ catalyst with the PPh_3_ ligand produced high-molecular-weight polymers in high polymerization yields without any co-ligands. A large excess of PPh_3_ is recommended to achieve a high polymerization yield and high molecular weight [21]. Even moderate equivalents of PPh_3_ afforded good yields. Consequently, polymerization under optimized conditions not only provided a high-molecular-weight block copolymer, but also high reproducibility. 

#### 3.2.1. Effect of the Equivalents of the Catalyst and Monomers on the Polymer Molecular Weight

**Figure 2:**
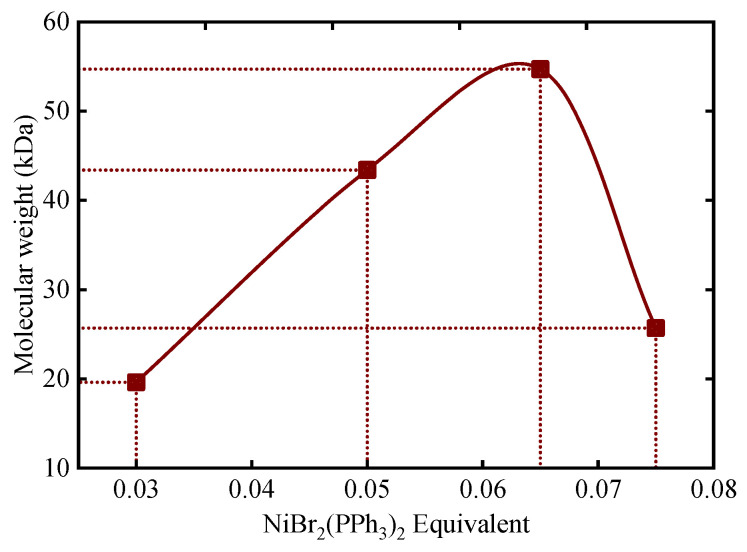
Effect of NiBr_2_(PPh_3_)_2_ equivalents on the sPP block copolymer molecular weight, when catalyst to ligand, zinc, and NaI ratios were maintained at 1:10, 1:30, and 1:1, respectively.

The effects of the equivalents of NiBr_2_(PPh_3_)_2_ on the polymerization of neopentyl-protected dichlorobenzene sulfonate monomers and Cl-end-capped OAEK were investigated. The molar equivalents of the feed comonomers (*p*NDCBS and *m*NDCBS) were kept constant at 1:1. The poly(*para*-phenylene) structure is highly rigid with a linear main chain, which lowers the solvent solubility, thereby inhibiting the molecular weight build-up. Feeding an equivalent molar ratio of *meta*-monomers is an effective and promising approach to improve solvent solubility and the molecular weight of the polymer. The reaction conditions and GPC results are summarized in Table 1, and the corresponding GPC profiles are shown in Figure S2. The dependence of the NiBr_2_(PPh_3_)_2_ equivalents on the resultant polymer molecular weight is shown in Figure 2. Using 0.03 NiBr_2_(PPh_3_)_2_ equivalents afforded the block copolymer sPP30@1:1 with the lowest molecular weight (*M*_w_ = 20 kDa). The molecular weight of the polymer increased with increasing catalyst equivalent weight. The use of 0.065 equivalents afforded 55 kDa of the resultant block copolymer sPP65@1:1 in 92% yield; this polymer had good solubility in polar organic solvents. Therefore, we used higher equivalents to obtain a higher-*M*_w_ block copolymer. However, when 0.075 equivalents were used, the *M*_w_ of sPP75@1:1 (*M*_w_ = 26 kDa) decreased. This result is consistent with the results of a study by Colon and Kwiatkowski, where they observed that both high and low catalyst equivalents do not yield high-molecular-weight polymers [22]. Low-molecular-weight block copolymers are obtained because when most aryl chlorides react with Ni(0), the remaining Ni(0) forms aryl-nickel species without further reaction. We observed that 0.065 equivalents of NiBr_2_(PPh_3_)_2_ is the optimal amount of catalyst for the polymerization of sulfonated polyphenylenes to yield a high-molecular-weight block copolymer, considering the abovementioned monomers and oligomer.

**Figure 3:**
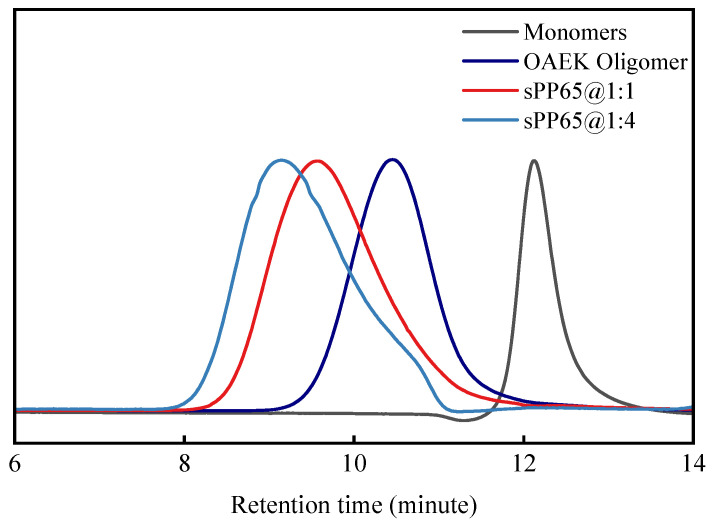
GPC profiles of the starting materials and sPP65@1:1 and sPP65@1:4 block copolymers catalyzed by NiBr_2_(PPh_3_)_2_ equivalents using different molar ratios of *p*NDCBS and *m*NDCBS; related properties are listed in Table 1.

The effect of the feed monomer ratio on the molecular weight of the block copolymers was also investigated. Polymerization was conducted under previously optimized conditions using a 1:4 ratio of *p*NDCBS and *m*NDCBS monomers. The GPC profiles of the sPP65@1:1 and sPP655@1:4 block copolymers are shown in Figure 3, and the quantitative GPC results are summarized in Table 1. The reactivity of less-sterically hindered compounds is much higher than that of sterically hindered compounds. When high equivalents of *para*-substituted monomer are used in block copolymerization, the resultant block copolymer has a linear and rigid main chain, which lowers the solubility and limits the molecular weight [23]. The flexibility of the main chain in the sPP65@1:4 block copolymer is the governing factor in achieving a high molecular weight. Generally, Ni-catalyzed coupling block copolymers exhibit a high polydispersity index (PDI) [24]; however, in this study, both sPP65@1:1 and sPP65@1:4 block copolymers had relatively low PDI values, which implies that the in-situ-generated Ni(0) was highly reactive in the block copolymer synthesis. 

#### 3.2.2. Role of Zn and NaI in the Polymerization Reaction

**Scheme 2:**
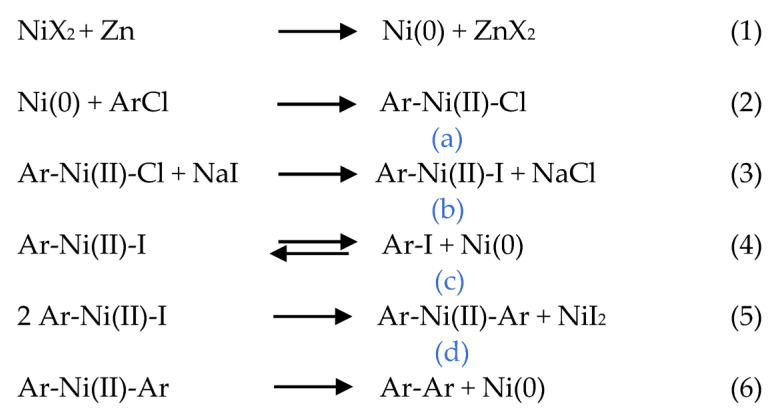
Reaction path demonstrating the role of Zn and NaI in Ni-catalyzed polymerization.

The mechanism of our reaction system is as follows: in-situ-generated Ni(0) from the Ni(II) salt in the presence of Zn actively reacts with Cl-end-capped OAEK and neopentyl-protected dichloro monomers to yield an oxidative addition adduct (a) (Scheme 2). In the presence of NaI, adduct (a) undergoes metathetical displacement to yield another oxidative adduct (b). Subsequently, adduct (b) readily produces adducts (c) and (d). In addition, disproportionation of adduct (b) yields adduct (d), which subsequently produces a high-molecular-weight polymer. The polymerization reaction involves a sequence of oxidative addition, metathesis, and reductive elimination steps, which are well known in transition-metal-catalyzed chemistry. To guarantee efficient Ni(0)-catalyzed polymerization, Ni(0) must be regenerated from the Ni(II) species formed during the reaction; in this reaction, Zn and NaI aid in the regeneration of the Ni(0) species. The use of NaI has a positive effect on polymerization [16,20]. Earlier studies on biaryl synthesis postulated that the presence of alkali metal salt additives (iodide or bromide salts) accelerates the coupling reaction [17,25]. However, these studies focused only on synthesizing biaryl compounds, rather than high-molecular-weight polyphenylenes. 

#### 3.2.3. Block Copolymer Synthesis Using Ni(COD)_2_

For comparison, sPP(COD)@1:1 and sPP(COD)@1:4 block copolymers were synthesized using the Ni(COD)_2_ catalyst using the molar equivalents of 1:1 and 1:4 of *p*NDCBS to *m*NDCBS, respectively. To catalyze polymerization with Ni(COD)_2_, a very large amount of 1.25 equivalents to those of total chlorine was used. This is because the Ni(II) species formed in the oxidative addition step could not be reduced to Ni(0) in situ. The GPC profiles of sPP(COD)@1:1 and sPP(COD)@1:4 are shown in Figure S2, and the properties associated with the data are listed in Table 1. Although the molecular weights of the block copolymers synthesized with the Ni(COD)_2_ catalyst were slightly lower than those of the block copolymers synthesized with the NiBr_2_(PPh_3_)_2_ catalyst, all polymers not only had high molecular weights, which were sufficient for casting membranes, but also demonstrated good solubility in polar organic solvents. 

### 3.3. Structural Characterization of the Synthesized Block Copolymers

**Figure 4:**
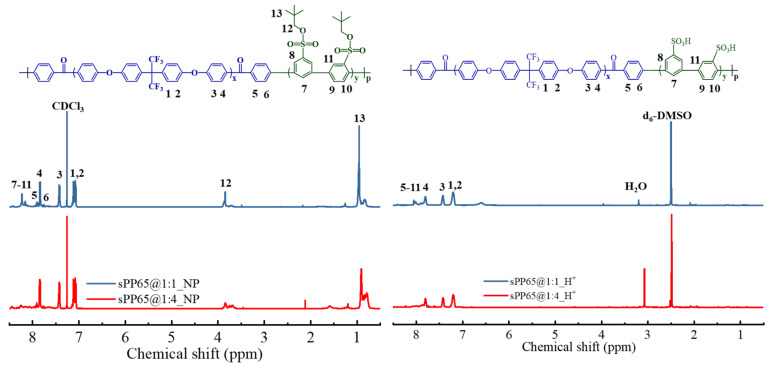
^1^H NMR spectra of sPP65@1:1 and sPP65@1:4 block copolymers in the neopentyl-protected and protonated forms recorded in CDCl_3_ and *d*_6_-DMSO, respectively.

The ^1^H NMR spectra of the block copolymers synthesized in this study are shown in Figure S3 and Figure 4. The chemical shift values for all block copolymers were consistent with the predicted values of the proposed structures. Moreover, the chemical shifts of the neopentyl group indicated that it was preserved after block copolymerization. The ^1^H NMR spectra in Figure 4 suggests a complete deprotection step because the neopentyl group was not observed. Furthermore, the solubility of the block copolymers remained unchanged even after deprotection. The ^1^H NMR spectra also shows that the chemical shifts corresponding to the aromatic region remained intact. However, the chemical shifts of the OAEK repeating unit remained identical to those of the sPP block copolymers, whereas those of the end group protons shifted downfield in the block copolymer.

### 3.4. Fabrication of PEMs from the Synthesized sPP Block Copolymers

**Figure 5:**
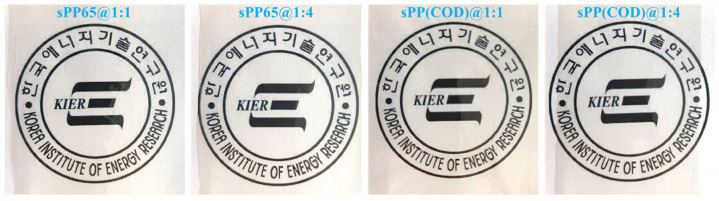
Digital photographs of sPP65 and sPP(COD) block copolymer membranes obtained from the polymerization of sPP65 block copolymers catalyzed using NiBr_2_(PPh_3_)_2_ and Ni(COD)_2_, respectively.

The block copolymers sPP65@1:1 and sPP65@1:4, which had the highest molecular weights, were selected for membrane casting. Thin, transparent, and bendable membranes were obtained from these copolymers using the solution-casting technique. Digital photographs of the fabricated membranes are shown in Figure 5. These results imply that the polymerization catalyzed by the in-situ-generated Ni(0) species from the Ni(II) salt in the presence of Zn was successful. For comparison, we also prepared membranes from the block copolymers synthesized using the Ni(COD)_2_ catalyst.

### 3.5. Ion Exchange Capacity, Proton Conductivity, Water Uptake, and Dimension Stability of Membranes

A feed IEC of 2.50 meq/g and a hydrophobic–hydrophilic block structural composition of X_5_Y_11.3_ were used for the block copolymer synthesis. The calculated IEC values for block copolymer membranes are listed in Table 2, and these values are in good agreement with the feed IEC values.

**Figure 6:**
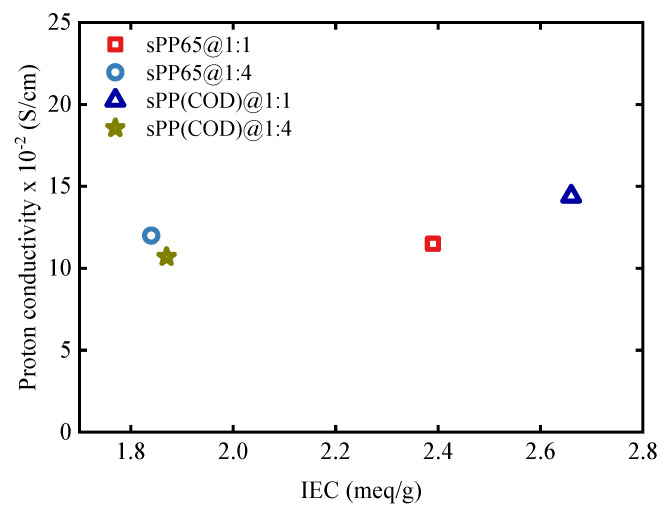
Proton conductivity of sPP block copolymer membranes measured in water at 25 °C.

Proton transport in PEMs occurs through water-filled percolating hydrophilic domains surrounding the acid functionalities, i.e., water uptake (*Φ*_w_) is a key property of proton-conducting membranes and directly influences proton conductivity. However, excessive water uptake under hydration conditions negatively impacts the mechanical and dimensional properties of the membrane. Table 2 lists the *Φ*_w_ values, which range between 61% and 71%. The water uptake consistently followed an increasing trend with an increase in relative humidity (Figure S4). Anisotropic dimensional variations were observed for all block copolymer membranes, where the Δ*t* values were greater than Δ*l* values (Table 2). This is attributed to the difference in chain alignment between the hydrophobic and hydrophilic segments in the block copolymer main backbone [8]. The rigid hydrophobic segment aligned in the in-plane direction, whereas the hydrophilic segment aligned in the through-plane direction. Hydrophilic moieties are solely responsible for membrane swelling under hydration conditions. Figure 6 shows the proton conductivities of the block copolymer membranes, which are in accordance with their IEC values. The conductivity values for the sPP65@1:4 copolymer membrane surpassed the conductivity value of sPP65@1:1 despite the low IEC value. This is attributed to the more flexible hydrophilic segment in sPP65@1:4, where the molar equivalent for the *meta*-comonomer is 4 equivalents to the *para*-comonomer.

## 4. Conclusions

In this study, we highlighted the potential of in-situ-generated Ni(0) in the presence of the strong reducing metal Zn and the weak additive ligand NaI as the polycondensation promoter in the synthesis of sulfonated polyphenylenes. The effects of Ni(II) complex equivalents to those of total chlorine and the hydrophilic monomer feed ratio of the resultant polymer were investigated. The in-situ-generated Ni(0) from Ni(II)Br_2_(PPh_3_)_2_ in the presence of Zn successfully catalyzed the synthesis of sulfonated polyphenylene block copolymers in catalytic equivalents. An increase in the NiBr_2_(PPh_3_)_2_ equivalents up to 0.065 equivalents afforded high-molecular-weight block copolymers. The resultant block copolymers synthesized using NiBr_2_(PPh_3_)_2_ had comparable physical properties and chemical structures to those synthesized using Ni(COD)_2_. The water sorption parameters and ion transport properties of the block copolymers sPP65@1:1 and sPP65@1:4, which had the highest molecular weights, were studied in detail. We believe that our approach will open new avenues for the synthesis of other functional block copolymers. 

## Figures and Tables

**Table 1:** Summary of feed ratios of monomers, catalysts, and ligands, GPC results, and yields of block copolymers.

Copolymer	Catalyst	Catalyst Equivalents ^a^	Ligand	Ligand Equivalents ^b^	Comonomer Molar Ratio ^c^	*M*_n_ (kDa)	*M*_w_ (kDa)	PDI	Yield
sPP30@1:1 sPP50@1:1 sPP65@1:1 sPP75@1:1 sPP65@1:4 sPP(COD)@1:1 sPP(COD)@1:4	NiBr_2_(PPh_3_)_2_ NiBr_2_(PPh_3_)_2_ NiBr_2_(PPh_3_)_2_ NiBr_2_(PPh_3_)_2_ NiBr_2_(PPh_3_)_2_ Ni(COD)_2_ Ni(COD)_2_	0.030 0.050 0.065 0.075 0.065 1.25 1.25	PPh_3_ PPh_3_ PPh_3_ PPh_3_ PPh_3_ BPy BPy	10 10 10 10 10 2 2	1:1 1:1 1:1 1:1 1:4 1:1 1:4	10 17 22 10 30 18 25	20 43 55 26 65 48 57	1.9 2.5 2.5 2.5 2.2 2.5 2.3	76 66 92 72 83 86 81

^a^ Molar ratio of the catalyst to the feed monomer. ^b^ Molar ratio of the ligand to the catalyst. ^c^ Molar equivalents of *p*NDCBS to *m*NDCBS. Abbreviations: M_n:_ number average molecular weight, M_w_: polymer molecular weight, and PDI: polydispersity index.

**Table 2:** Measured ion exchange capacity, water uptake (*Φ*_w_), and in-plane (Δ*l*) and through-plane (Δ*t*) swelling ratios for block copolymers selected for membrane casting.

Copolymer	IEC (meq/g)		*Φ*_w_ (%) 25 °C	Swelling Ratio (%) 25 °C	
	Feed	Measured ^a^		Δ*l*	Δ*t*
sPP65@1:1	2.50	2.39	63	22	34
sPP65@1:4	2.50	1.84	59	17	30
sPP(COD)@1:1	2.50	2.66	71	36	36
sPP(COD)@1:4	2.50	1.87	61	22	30

^a^ Calculated using ^1^H NMR data.

## Data Availability

The data presented in this study are available upon request from the corresponding authors.

## Supplementary Materials

The following supporting information can be downloaded at: https://www.mdpi.com/article/10.3390/polym15061577/s1, Figure S1: ^1^H-NMR spectra of 3,5-dichlorobenzene sulfonyl chloride (mDCBS), 1-neopentylsulfonyl-3,5-dichlorobenzene (mNDCBS), 2,5-dichlorobenzene sulfonyl chloride (pDCBS), and 1-neopentylsulfonyl-2,5-dichlorobenzene (pNDCBS) recorded in CDCl_3_ solution.; Figure S2: Gel permeation chromatography profiles of starting materials [pNDCBS, mNDCBS, and oligo(arylene ether ketone) (OAEK)] and sPP block copolymers catalyzed by different equivalents of NiBr_2_(PPh_3_)_2_ [sPP30@1:1, sPP50@1:1, and sPP75@1:1] and Ni(COD)_2_ [sPP(COD)@1:1 and sPP(COD)@1:4] using different molar ratios of pNDCBS and mNDCBS; Figure S3: ^1^H NMR spectra of block copolymers in their neopentyl-protected forms; Figure S4: Dependence of water vapor uptake of the sPP membranes on the relative humidity measured at 70 ℃.; Table S1: Summary of Ni(II)-salt-catalyzed polymerization in previous studies and current work. References [26,27] are cited in the supplementary materials.
