# Supplementary material for: Synthesis of Sulfonated Polyphenylene Block Copolymers via In Situ Generation of Ni(0)
Source: Polymers (Basel). 2023 Mar 22;15(6):1577. doi: 10.3390/polym15061577 (PMC10058434; doi:10.3390/polym15061577)
Supplement: Supplementary file 1 [file polymers-15-01577-s001.zip › polymers-2278478-supplementary.pdf]

Supporting Information

Synthesis of Sulfonated Polyphenylene Block Copolymers via In Situ Generation of Ni(0)

Vikrant Yadav [1], Farid Wijaya [1,2], Hyejin Lee [1], Byungchan Bae [1,2,*] and Dongwon Shin [1,2,*]

[1] Fuel Cell Laboratory, Korea Institute of Energy Research, Daejeon 34129, Republic of Korea; yadav.vikrant003@gmail.com (V.Y.); faridw@kier.re.kr (F.W.); hyejinlee@kier.re.kr (H.L.)
[2] Hydrogen Energy Engineering, University of Science and Technology, Daejeon 34113, Republic of Korea
* Correspondence: bcbae@kier.re.kr (B.B.); dwshin@kier.re.kr (D.S.)

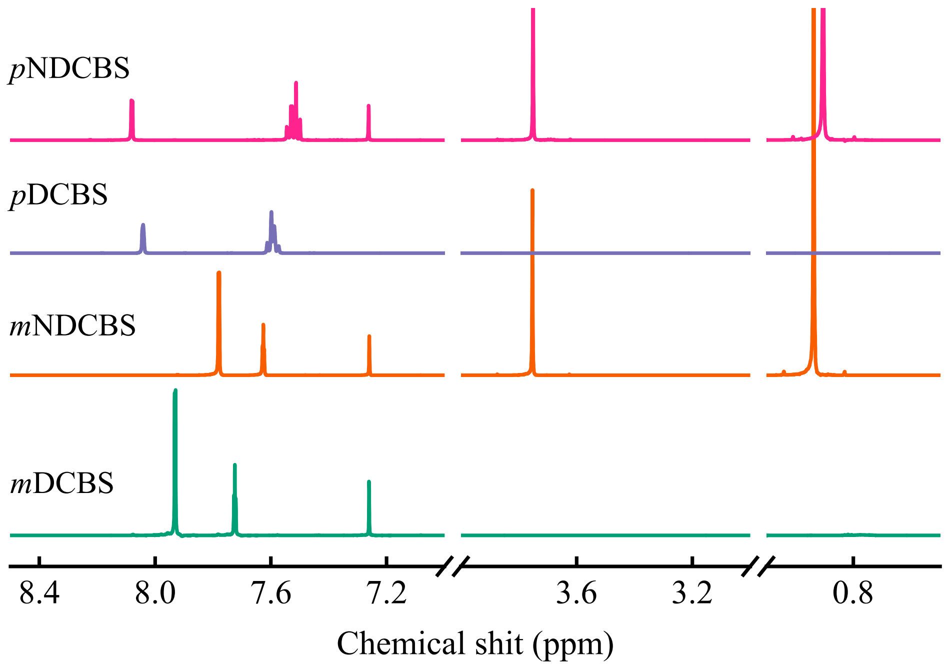

Figure S1. ^{1}H-NMR spectra of 3,5-dichlorobenzene sulfonyl chloride (mDCBS), 1-neopentylsulfonyl-3,5-dichlorobenzene (mNDCBS), 2,5-dichlorobenzene sulfonyl chloride (pDCBS), and 1-neopentylsulfonyl-2,5-dichlorobenzene (pNDCBS) recorded in $CDCl_3$ solution.

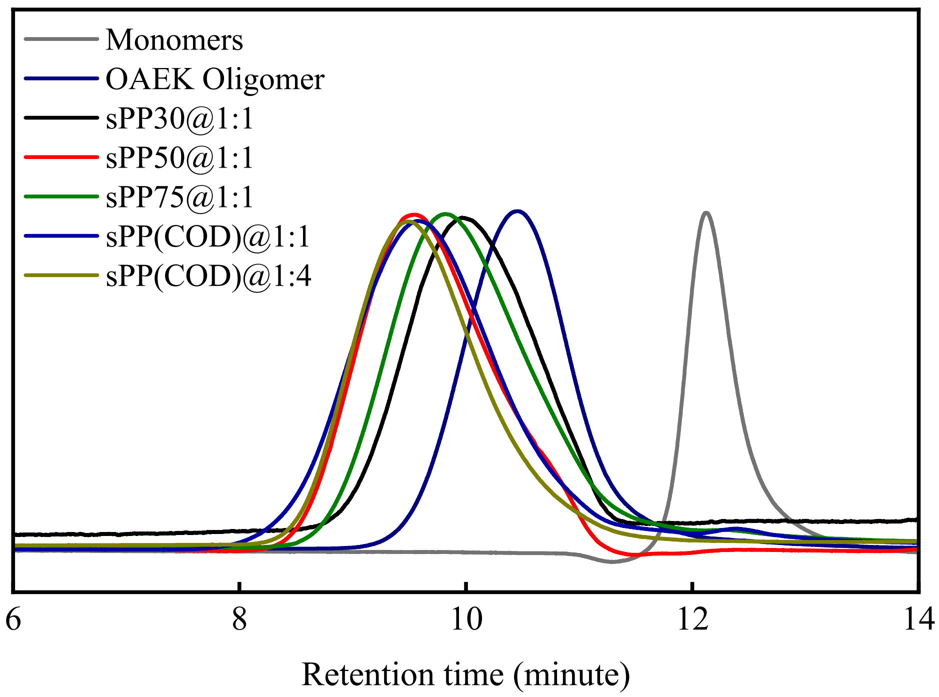

Figure S2. Gel permeation chromatography profiles of starting materials [pNDCBS, mNDCBS, and oligo(arylene ether ketone) (OAEK)] and sPP block copolymers catalyzed by different equivalents of $NiBr_2(PPh_3)_2$ [sPP30@1:1, sPP50@1:1, and sPP75@1:1] and $Ni(COD)_2$ [sPP(COD)@1:1 and sPP(COD)@1:4] using different molar ratios of pNDCBS and mNDCBS; some properties of these copolymers are summarized in Table 1.

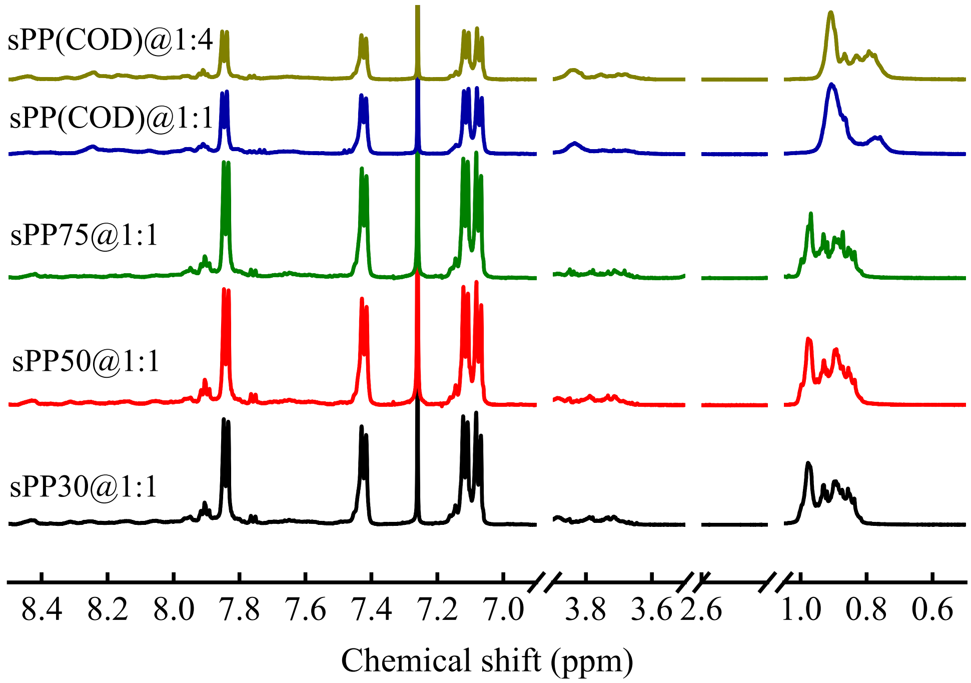

Figure S3. ^{1}H NMR spectra of block copolymers in their neopentyl protected forms recorded in $CDCl_3$.

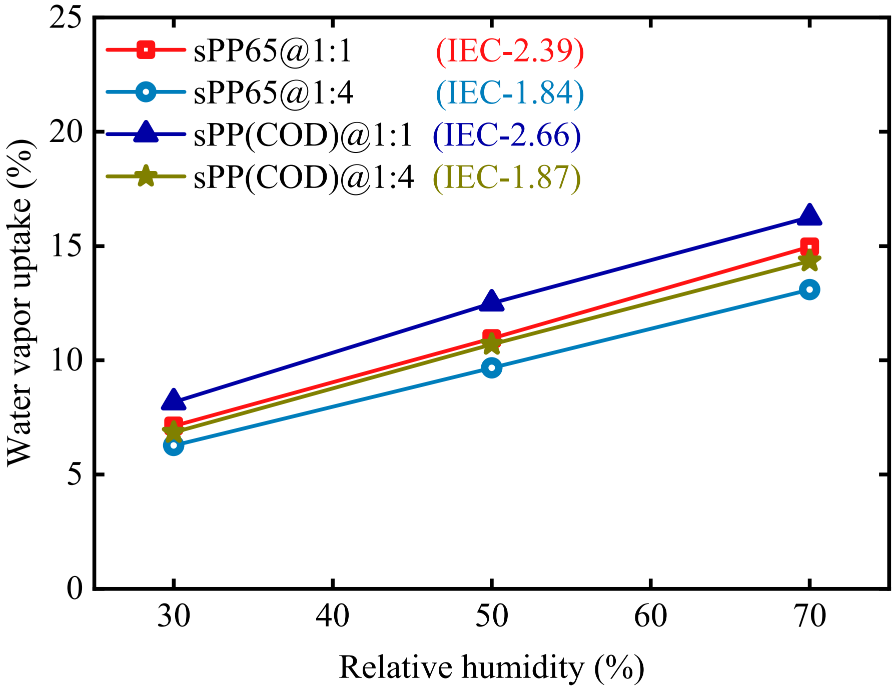

Figure S4. Dependence of water vapor uptake of the sPP membranes on the relative humidity measured at 70 °C.

Table S1. Summary of Ni(II)-salt-catalyzed polymerization in previous studies and current work

Poly(phenylene)s	Catalyst	Catalyst equivalents	Ligand used	Ligand equivalents	References
SFPP	$NiCl_2(PPh_3)_2$	0.030	PPh_3	13	1
PBP-Z	$NiCl_2(PPh_3)_2$	0.030	PPh_3	12	7
sPP-b-PES	$NiBr_2$	0.070	PPh_3	07	8
sPP-b-PKS	$NiBr_2$	0.070	PPh_3	07	8
sPP-b-PPSU	$NiBr_2$	0.070	PPh_3	07	8
sPP-b-PPES	$NiBr_2$	0.070	PPh_3	07	8
PTSP-b-PAESs	$NiBr_2$	0.200	PPh_3	10	9
PPBPs	$NiCl_2(PPh_3)_2$	0.033	PPh_3	12	12
PES	$NiCl_2$	0.050	PPh_3	24	21
PEEK	$NiCl_2$	0.050	PPh_3	02	26
PAPO	$NiCl_2$	0.050	PPh_3	02	27
sPP65@1:1	$NiBr_2(PPh_3)_2$	0.065	PPh_3	10	This work
sPP65@1:4	$NiBr_2(PPh_3)_2$	0.065	PPh_3	10	This work

SFPP - Poly(4-phenoxybenzoyl-1,4-phenylene) with 1,1,2,2-tetrafluoro-2-oxyethane-1-sulfonic acid; PBP-Z - poly(2,5-benzophenone-co-4'-fluorine-2,5-benzophenone); sPP-b-PES – No abbreviation provided; sPP-b-PKS - No abbreviation provided; sPP-b-PPSU - No abbreviation provided; sPP-b-PPES - No abbreviation provided; PTSP-b-PAESs - poly(tri-sulfonated phenylene)-block-poly(arylene ether sulfone) copolymers; PPBPs - poly(4-phenoxybenzoyl-1,4-phenylene)s; PES – poly(ether sulfone)s; PEEK - poly(ether ketone)s; PAPO - poly(arylene phosphine oxide).
